# Effects of trigger-day progesterone in c-IVF/ICSI cycles on blastocyst culture outcomes

**DOI:** 10.3389/fendo.2025.1496803

**Published:** 2025-02-12

**Authors:** Yating Sun, Jia Wang, Luyun Zhang, Yanjun Chang, Aizhen Zhu

**Affiliations:** Department of Reproductive Medicine, Yuncheng Central Hospital affiliated to Shanxi Medical University, Yuncheng, China

**Keywords:** conventional *in vitro* fertilization (c-IVF), intracytoplasmic sperm injection (ICSI), trigger-day progesterone (P), gonadotropin-releasing hormone (GnRH) antagonist, available blastocyst rate, top-quality blastocyst rate

## Abstract

**Objective:**

To assess whether trigger-day progesterone (P) levels in conventional *in vitro* fertilization (c-IVF)/intracytoplasmic sperm injection (ICSI) cycles are associated with blastocyst culture outcomes.

**Methods:**

In this retrospective analysis, 747 eligible patients (747 cycles) who adopted the gonadotropin-releasing hormone (GnRH) antagonist protocol and underwent c-IVF/ICSI between January 2021 to June 2024 were recruited. The P cutoff values were 1.0 and 1.5 ng/ml when trigger-day serum P was measured, and 4177 day3 (D3) embryos for blastocyst culture were grouped according to trigger-day P levels. Furthermore, the effects of trigger-day P on blastocyst culture outcomes were evaluated.

**Results:**

In total, 747 cycles, 4177 D3 embryos for blastocyst culture were analyzed. After adjustments, multivariate logistic regression analysis revealed that compared with those in the normal level group, available blastocyst rate (adjusted OR, 0.780; 95% CI, 0.645-0.942; P=0.010) and D5 available blastocyst rate (adjusted OR, 0.736; 95% CI, 0.604-0.898; P=0.003) in the high level group were significantly reduced. Subgroup analysis showed that when female age was less than 35 years old, compared with that (36.30%) in the normal level group, the D5 available blastocyst rate (36.92%, adjusted OR, 0.744; 95% CI, 0.602-0.920; P=0.006) in the high level group was significantly reduced. In ICSI cycles, compared with that (28.69%) in the normal level group, the D5 available blastocyst rate (19.13%, adjusted OR, 0.369; 95% CI, 0.194-0.703; P=0.002) in the high level group was significantly decreased.

**Conclusion(s):**

This study demonstrated that in the c-IVF/ICSI population, the trigger-day slightly elevated P (1.0-1.5ng/ml) was not related to blastocyst culture outcomes, while the trigger-day elevated P (>1.5ng/ml) was an important factor affecting D5 available blastocyst rate, especially when the woman was younger than 35 years old or insemination type was ICSI.

## Introduction

With the maturity of embryo culture technology, many reproductive centers will carry out blastocyst culture for day 3 (D3) available cleavage-stage embryos cultured *in vitro*. By prolonging the embryo culture time, embryos with low developmental potential or genetic defects will be eliminated to a certain extent, and embryos with more developmental potential will be further screened out (1). Blastocyst transfer also improves the endometrial receptivity, increases the chance of implantation, improves the clinical pregnancy rate, and reduces the occurrence of multiple pregnancies, premature births and low birth weight infants (2–5). However, not all patients are suitable for blastocyst culture. Blastocyst culture has a certain risk that some embryos will be eliminated during the culture process, resulting in fewer or no available blastocysts for patients. At present, there is a lack of effective indicators to accurately predict the outcomes of blastocyst culture.

A large number of studies have confirmed that during conventional *in vitro* fertilization (c-IVF)/intracytoplasmic sperm injection (ICSI) controlled ovarian stimulation (COS), trigger-day elevated progesterone (P) not only reduces endometrial receptivity (6–8), advances the implantation window (9), and leads to lower clinical pregnancy rates, ongoing pregnancy rates, and live birth rates (10–21), but also affects embryo quality (6, 19, 22). However, there is still insufficient evidence on whether trigger-day elevated P level will also affect the outcomes of blastocyst formation on day 5 (D5) or day6 (D6) and increase the risk of no available blastocyst. The existing few studies are also partial, including only ICSI/preimplantation genetic testing (PGT) cycles or only top-quality blastocyst formation rate (6). Therefore, the purpose of this study was to evaluate the effects of trigger-day elevated P on available blastocyst rate, top-quality blastocyst rate and blastocyst formation time in c-IVF/ICSI cycles to provide reference for assisted reproductive technology (ART) clinical work.

## Materials and methods

### Study design and population

This retrospective cohort study included 1095 c-IVF/ICSI cycles of COS with gonadotropin-releasing hormone (GnRH) antagonist protocol from January 2021 to June 2024. The cycle exclusion criteria were as follows: The women over 40 years old; Chromosomal abnormalities in the male or female partners; More than 2 c-IVF/ICSI cycles; Missed relevant data; Low ovarian response. Oocytes or day3 cleavage-stage embryos (D3 embryos) those met any of the following criteria were excluded: Abnormal fertilized or unfertilized oocytes; Unavailable D3 embryos; Frozen or transplanted D3 embryos. Ultimately, **747** cycles (**747** eligible patients), with a total of 4177 D3 embryos for blastocyst culture were included in this study. Owing to the retrospective nature of the study, informed consent was waived. All operations were carried out in conformity with the applicable rules and regulations.

As described in a prior study (23), patients adopted the same protocol, namely, GnRH antagonist protocol. The dosage of gonadotropin (Gn) was individually coordinated according to the basic characteristics and responses of each patient. Continuous transvaginal ultrasound scans and serum estradiol (E_2_), luteinizing hormone (LH) and P were used to track the cycles. Triggering was employed with 250 µg recombinant human chorionic gonadotropin (r-hCG, Merck Serono, Geneva, Switzerland) and 3,000 IU u-HCG (Livzon, Guangzhou, China). Thirty-six hours later, oocytes were retrieved under the guidance of transvaginal ultrasound and cultured in incubator for 2 ~ 4 hours prior to fertilization.

### Laboratory procedures

Based on the total progressively motile sperm cell count (TPMC) after semen treatment on the day of oocyte retrieval, c-IVF (TPMC≥5 million) or ICSI (TPMC < 5 million) insemination method was selected. Fertilization was observed 20h after c-IVF or 17h after ICSI. The D3 embryos were scored on the third day after oocyte retrieval (24). Grade I, grade II and grade III embryos were defined as available embryos, Grade IV embryos were defined as unavailable embryos (discarded embryos). According to the condition of the patients, the D3 available embryos could be frozen, transferred or continued to culture until D5 or D6. If blastocyst culture was performed, the blastocysts were scored on D5 or D6 after oocyte retrieval based on previous literature (25, 26). The available blastocysts were considered as an expanded, hatching, or hatched blastocyst with ICM/TE grading (AA, AB, BA, BB, AC, CA, BC, CB, and CC) in this study. The top-quality blastocysts were considered as an expanded, hatching, or hatched blastocyst with high ICM/TE grading (AA, AB, BA, and BB).

### P assessment

The sex hormones (E_2_, LH, P) concentrations were measured from 8:00 am to 9:00 am on the trigger-day by siemens automatic chemiluminescence immunoanalyzer (ADVIA Centaur CP). The detection limit of chemiluminescence immunoassay was 0.03 ng/mL, the sensitivity was 0.15 ng/ml, the coefficient of variation within the group was 3.0%, and the coefficient of variation between the groups was 5.5%. The same detection method was used throughout the study and calibrated regularly to reduce unnecessary errors.

### Main outcome measures

The key result of the study was D5 available blastocyst rate, with other indicators being D6 available blastocyst rate and top-quality blastocyst rate. Available blastocyst rate was defined as number of available blastocysts per cultured, top-quality blastocyst rate was defined as number of top-quality blastocysts per cultured (11).

### Statistical analysis

Statistical analysis was performed using SPSS Statistics for Windows version 26. Trigger-day P was regarded as a categorical variable, cycles were divided into the following three groups according to trigger-day P levels: The normal level group included cycles in which trigger-day P were less than 1.0ng/ml; The slightly elevated level group included cycles in which trigger-day P were between 1.0 ~ 1.5 ng/ml; The High level group included cycles in which trigger-day P were more than 1.50 ng/ml. Currently, there was no clear cut-off value for trigger-day P, so these cut-off values were chosen based on clinical practice. Trigger-day elevated P (>1.5 ng/ml) may affect embryo quality (11, 19). Additionally, the physiological P range is generally believed to be less than 1.0ng/ml. And in a retrospective analysis in 2022 (27), Wei et al. also set the threshold of slightly elevated P at 1.0ng/ml. Therefore, 1.0 ng/mL and 1.50 ng/ml were selected as the cut-off values of slightly elevated P level and high P level, respectively. After analysis by Kolmogorov-Smirnov method, all the continuous variables did not conform to normal distribution, which were presented as interquartile interval and were compared between groups using the Kruskal-Wallis test. We used frequencies (percentages) to represent categorical variables and used pearson chi-square test or likelihood ratio test to analyze the differences between groups. Multivariate logistic regression model was established to calculate the adjusted relative risks (RRs) and 95% confidence intervals (CIs). Potential confounders were selected according to routine clinical practice, existing literature reports (28, 29) and variables with significant differences (P<0.05) in the univariate analysis, and adjustments were made for these confounding variables in the analysis of changes in each outcome between groups to explore the relationship between trigger-day P and blastocyst culture outcomes. Adjusted confounding variables included female age, male age, infertility duration, infertility factors, female body mass index (BMI), total antral follicle count (AFC), cycle number, Gn total dosage, trigger-day E_2_ level, trigger-day LH level, dominant follicle number, insemination type, D3 embryo numbers for blastocyst culture. All tests were bilateral, and statistical significance was defined as P<0.05.

## Results

### Patient demographics and general characteristics

**Figure 1:**
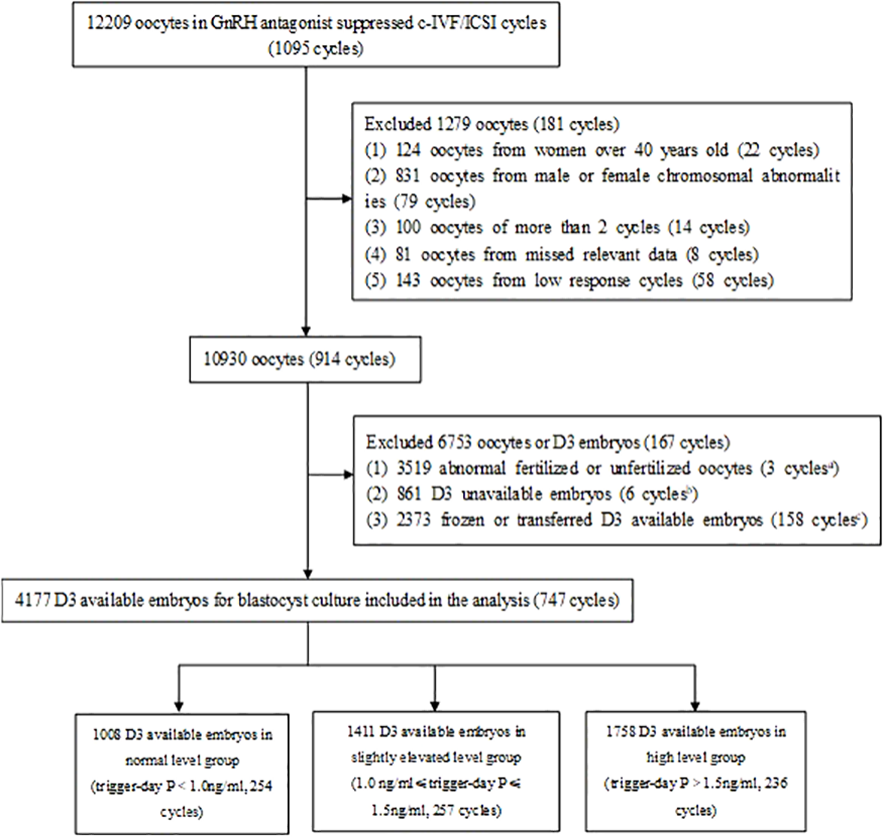
Study flowchart. GnRH, gonadotropin-releasing hormone; c-IVF, conventional in vitro fertilization; ICSI, intracytoplasmic sperm injection, D3, day 3; P, progesterone. 3 cycles in which all oocytes were abnormal fertilized or unfertilized were excluded. 6 cycles in which all D3 embryos were unavailable were excluded. 158 cycles in which all D3 available embryos were frozen or transferred were excluded.

A total of 1095 cycles (12209 oocytes) underwent GnRH antagonist protocol for controlled ovarian stimulation and adopted c-IVF/ICSI. Among them, 22 cycles (124 oocytes) with women over 40 years old, 79 cycles (831 oocytes) with male or female chromosomal abnormalities, 14 cycles (100 oocytes) with more than 2 cycles, 8 cycles (81 oocytes) with missed relevant data and 58 cycles (143 oocytes) with low response were excluded. In 10930 oocytes (914 cycles), 3 519 abnormal fertilized or unfertilized oocytes (3 cycles), 861 D3 unavailable embryos (6 cycles), 2373 frozen or transferred D3 embryos (158 cycles) were excluded. Finally, 4177 D3 embryos for blastocyst culture (**747** cycles) were included in the analysis (**Figure 1**).

Among them, There were 1008 D3 embryos (254 cycles) in normal trigger-day P group (P<1.0 ng/ml), 1411 D3 embryos (257 cycles) in slightly elevated trigger-day P group (1.0≤P ≤ 1.5 ng/ml), and 1758 D3 embryos (236 cycles) in high trigger-day P group (P>1.5 ng/ml) (**Figure 1**, **Table 1**). There were no significant differences in the following indicators grouped by trigger-day P: female age, male age, infertility duration, type of infertility, infertility factors, gravidity, parity and miscarriage (P>0.05). Female BMI (24.0 vs. 23.4 vs. 23.2, P=0.019) significantly decreased with increasing trigger-day P, total antral follicle count (AFC, 12 vs. 14 vs. 18) and the proportion of the first cycles (84.65% vs. 93.00 vs. 94.07, P<0.001) significantly increased with increasing trigger-day P (**Table 1**).

**Table 1:** Patient and blastocyst characteristics in c-IVF/ICSI cycles included.

Characteristics	Trigger-day P level (ng/ml)			*P* value
	Normal level group (<1.0)	Slightly elevated level group (1.0-1.5)	High level group (>1.5)	
No. of cycles	254	257	236	
No. of D3 available embryos for blastocyst culture	1008	1411	1758	
Female age, median (IQR), y	30(28,33)	30(27,33)	29(27,32)	0.062a
Male age, median (IQR), y	31(29,34)	31(29,34)	30(28,34)	0.109a
Infertility duration, median (IQR), y	3(2,5)	3(2,5)	3(2,5)	0.841a
Type of Infertility, n(%)				0.378b
Primary	125(49.21)	134(52.14)	131(55.51)	
Secondary	129(50.79)	123(47.86)	105(44.49)	
Infertility factors, n(%)				0.272b
tubal factor	134(52.76)	157(61.09)	140(59.32)	
Ovulation disorders and low ovarian reserve	74(29.13)	51(19.84)	58(24.58)	
Male factor	25(9.84)	25(9.73)	17(7.20)	
Others	21(8.27)	24(9.34)	21(8.90)	
Gravidity, n(%)				0.405b
0	123(48.43)	126(49.03)	126(53.39)	
1	71(27.95)	70(27.24)	49(20.76)	
2	60(23.62)	61(23.74)	61(25.85)	
Parity, n(%)				0.079c
0	204(80.32)	204(79.38)	194(82.20)	
1	44(17.32)	49(19.07)	42(17.80)	
2	6(2.36)	4(1.55)	0(0.00)	
Miscarriage, n(%)				0.278b
0	183(72.05)	181(70.43)	176(74.58)	
1	46(18.11)	60(23.35)	41(17.37)	
2	25(9.84)	16(6.22)	19(8.05)	
Female BMI, median (IQR), kg/m2	24.0(21.6,27.1)	23.4(21.5,26.0)	23.2(20.8,26.3)	0.019a,d
Total antral follicle count(AFC), median (IQR)	12(9,19)	14(10,20)	18(12,24)	<0.001a,d
Cycle number, n(%)				<0.001b,d
1	215(84.65)	239(93.00)	222(94.07)	
2	39(15.35)	18(7.00)	14(5.93)	
Gn total dose,median (IQR), U	2475(1800,3075)	2400(1725,3000)	2250(1650,3075)	0.277a
Dominant follicle number, median (IQR)	8(6,10)	9(7,13)	11.5(9,15)	<0.001a,d
Trigger-day E2 level, median (IQR), pg/ml	2895(2176,3769)	3697(2890,5188)	5748(4282,8209)	<0.001a,d
Trigger-day LH level, median (IQR), U/L	1.88(1.27,2.76)	1.92(1.09,3.25)	1.72(0.91,2.95)	0.176a
Trigger-day P level, median (IQR), ng/ml	0.75(0.59,0.87)	1.22(1.10,1.36)	1.90(1.69,2.35)	<0.001a,d
Insemination type, n(%)				0.059b
Conventional IVF	186(73.23)	199(77.43)	194(82.20)	
ICSI	68(26.77)	58(22.57)	42(17.80)	
Available blastocyst rate	478/1008(47.42)	678/1411(48.05)	868/1758(49.37)	0.572b
D5	359/1008(35.62)	512/1411(36.29)	637/1758(36.23)	0.933b
D6	119/1008(11.80)	166/1411(11.76)	231/1758(13.14)	0.420b
Top-quality blastocyst rate	107/1008(10.62)	174/1411(12.33)	205/1758(11.66)	0.430b
D5	107/1008(10.62)	167/1411(11.83)	193/1758(10.98)	0.605b
D6	0/1008(0.00)	7/1411(0.50)	12/1758(0.68)	0.036b,d

c-IVF, conventional in vitro fertilization; ICSI, intracytoplasmic sperm injection; D3, day 3; Others, uterine factors or unexplained infertility; BMI, body mass index; AFC, antral follicle count; Gn, gonadotropin; E2, estradiol; LH, luteinizing hormone; P, progesterone; D5, day 5; D6, day 6.

Continuous variables are presented as median and (inter quartiles range).

a Kruskal-Wallis test;

b Pearson Chi-Square test;

c Likelihood-ratio test;

d Statistically significant values.

### Evaluations of differences among groups

The results indicated that the dominant follicle number and trigger-day E_2_ level were significantly increased respectively with increasing trigger-day P (P<0.05). Gn total dosage, trigger-day LH level, insemination type were no significant differences between groups (P>0.05, **Table 1**).

As shown in **Table 1**, There were no significant differences in the available blastocyst rate (47.42% vs. 48.05% vs. 49.37%, P=0.572), D5 available blastocyst rate (35.62% vs. 36.29% vs. 36.23%, P=0.933), D6 available blastocyst rate (11.80% vs. 11.76% vs. 13.14%, P=0.420), top-quality blastocyst rate (10.62% vs. 12.33% vs. 11.66%, P=0.430) and D5 top-quality blastocyst rate (10.62% vs. 11.83% vs. 10.98%, P=0.605) among the three groups. D6 top-quality blastocyst rate (0.00 vs. 0.50 vs. 0.68, P=0.036) were significantly increased respectively with increasing trigger-day P.

### Association between trigger-day P level and blastocyst culture outcomes

In the multivariate logistic regression model, compared with those in the normal level group, available blastocyst rate (adjusted OR, 0.864; 95% CI, 0.726-1.029; P=0.100), D5 available blastocyst rate (adjusted OR, 0.845; 95% CI, 0.704-1.015; P=0.072), D6 available blastocyst rate (adjusted OR, 1.003; 95% CI, 0.768-1.311; P=0.980), top-quality blastocyst rate (adjusted OR, 1.004; 95% CI, 0.763-1.323; P=0.975), D5 top-quality blastocyst rate (adjusted OR, 0.937; 95% CI, 0.710-1.238; P=0.649) in the slightly elevated level group were no significant change; D6 available blastocyst rate (adjusted OR, 1.064; 95% CI, 0.799-1.417; P=0.670), top-quality blastocyst rate (adjusted OR, 0.859; 95% CI, 0.635-1.161; P=0.323) and D5 top-quality blastocyst rate (adjusted OR, 0.787; 95% CI, 0.579-1.069; P=0.126) in the high level group were also no significant change; available blastocyst rate (adjusted OR, 0.780; 95% CI, 0.645-0.942; P=0.010) and D5 available blastocyst rate (adjusted OR, 0.736; 95% CI, 0.604-0.898; P=0.003) in the high level group were significantly reduced (**Table 2**).

**Table 2:** Adjusted blastocyst culture outcomes in c-IVF/ICSI cycles stratified by trigger-day P level (ng/ml).

Variables	Adjusted RR(95% CI)	*P* value
Available blastocyst rate		0.036a
Normal level group (<1.0)	Ref	Ref
Slightly elevated level group (1.0-1.5)	0.864(0.726-1.029)	0.100
High level group (>1.5)	0.780(0.645-0.942)	0.010a
D5 available blastocyst rate		0.010a
Normal level group (<1.0)	Ref	Ref
Slightly elevated level group (1.0-1.5)	0.845(0.704-1.015)	0.072
High level group (>1.5)	0.736(0.604-0.898)	0.003a
D6 available blastocyst rate		0.863
Normal level group (<1.0)	Ref	Ref
Slightly elevated level group (1.0-1.5)	1.003(0.768-1.311)	0.980
High level group (>1.5)	1.064(0.799-1.417)	0.670
Top-quality blastocyst rate		0.394
Normal level group (<1.0)	Ref	Ref
Slightly elevated level group (1.0-1.5)	1.004(0.763-1.323)	0.975
High level group (>1.5)	0.859(0.635-1.161)	0.323
D5 top-quality blastocyst rate		0.233
Normal level group (<1.0)	Ref	Ref
Slightly elevated level group (1.0-1.5)	0.937(0.710-1.238)	0.649
High level group (>1.5)	0.787(0.579-1.069)	0.126
D6b top-quality blastocyst rate	/	/

The multivariate regression model was adjusted for female age, male age, infertility duration, infertility factors, female BMI, AFC, cycle number, Gn total dosage, trigger-day E_2_ level, trigger-day LH level, dominant follicle number, insemination type, D3 embryo numbers for blastocyst culture.

P, progesterone; RR, relative risk; CI, confidence interval; Ref, reference; BMI: body mass index; AFC, total antral follicle count; Gn, gonadotropin; E_2_, estradiol; LH, luteinizing hormone; D3: day 3; D5, day 5; D6, day 6.

a Statistically significant values.

b D6 top-quality blastocyst numbers could not meet the requirement of sample size for multivariate regression analysis, so D6 top-quality blastocyst rate be unadjusted.

Then, we carried out subgroup analysis of adjusted D5 available blastocyst rate in c-IVF/ICSI cycles based on female age (<35 vs ≥35 years) and insemination type (c-IVF vs ICSI). When female age was less than 35 years old, the D5 available blastocyst rate was 36.69%. Compared with that (36.30%) in the normal level group, the D5 available blastocyst rate (36.67%, adjusted OR, 0.841; 95% CI, 0.693-1.021; P=0.081) in the slightly elevated level group had no significant change, which (36.92%, adjusted OR, 0.744; 95% CI, 0.602-0.920; P=0.006) in the high level group was significantly reduced. When female age was 35 years old or more, the D5 available blastocyst rate was 31.26%, which (33.70%, adjusted OR, 1.019; 95% CI, 0.556-1.867; P=0.951; 28.86%, adjusted OR, 0.772; 95% CI, 0.394-1.514; P=0.452) in the slightly elevated level group and the high level group had no significant change compared with that (30.58%) in the normal level group. In c-IVF cycles, the D5 available blastocyst rate was 38.77%, which (39.32%, adjusted OR, 0.916; 95% CI, 0.749-1.120; P=0.393; 38.81%, adjusted OR, 0.820; 95% CI, 0.662-1.015; P=0.069) in the slightly elevated level group and the high level group had no significant change compared with that (37.83%) in the normal level group. In ICSI cycles, the D5 available blastocyst rate was 22.98%. And compared with that (28.69%) in the normal level group, the D5 available blastocyst rate (20.78%, adjusted OR, 0.675; 95% CI, 0.413-1.105; P=0.118) in the slightly elevated level group had no significant change, which (19.13%, adjusted OR, 0.369; 95% CI, 0.194-0.703; P=0.002) in the high level group was significantly decreased (**Table 3**).

**Table 3:** Subgroup analysis of D5 available blastocyst rate in c-IVF/ICSI cycles stratified by trigger-day P level (ng/ml).

Variables	n (%)	*P* value^a^	Adjusted	
			RR (95% CI)	*P* value
Women,s age				
<35y	1367/3726(36.69)	0.954		0.024^b^
Normal level group (<1.0)	322/887(36.30)		Ref	Ref
Slightly elevated level group (1.0-1.5)	451/1230(36.67)		0.841(0.693-1.021)	0.081
High level group (>1.5)	594/1609(36.92)		0.744(0.602-0.920)	0.006^b^
≥35y	141/451(31.26)	0.629		0.653
Normal level group (<1.0)	37/121(30.58)		Ref	Ref
Slightly elevated level group (1.0-1.5)	61/181(33.70)		1.019(0.556-1.867)	0.951
High level group (>1.5)	43/149(28.86)		0.772(0.394-1.514)	0.452
Insemination type				
Conventional IVF	1346/3472(38.77)	0.803		0.167
Normal level group (<1.0)	289/764(37.83)		Ref	Ref
Slightly elevated level group (1.0-1.5)	464/1180(39.32)		0.916(0.749-1.120)	0.393
High level group (>1.5)	593/1528(38.81)		0.820(0.662-1.015)	0.069
ICSI	162/705(22.98)	0.029^b^		0.010^b^
Normal level group (<1.0)	70/244(28.69)		Ref	Ref
Slightly elevated level group (1.0-1.5)	48/231(20.78)		0.675(0.413-1.105)	0.118
High level group (>1.5)	44/230(19.13)		0.369(0.194-0.703)	0.002^b^

The multivariate regression model was adjusted for female age, male age, infertility duration, infertility factors, female BMI, AFC, cycle number, Gn total dosage, trigger-day E_2_ level, trigger-day LH level, dominant follicle number, insemination type, D3 embryo numbers for blastocyst culture.

P, progesterone; RR, relative risk; CI, confidence interval; Ref, reference; BMI: body mass index; AFC, total antral follicle count; Gn, gonadotropin; E_2_, estradiol; LH, luteinizing hormone; D3: day3; D5, day 5.

a Pearson Chi-Square test;

b Statistically significant values.

## Discussion

The results of our study showed that the trigger-day slightly elevated P (1.0-1.5ng/ml) was not related to blastocyst culture outcomes, while the trigger-day elevated P (>1.5ng/ml) was an important factor affecting D5 available blastocyst rate, especially when the woman was younger than 35 years old or insemination type was ICSI.

This study showed that there was no statistical difference in the D6 available blastocyst rate between different trigger-day P level groups. And after adjusting for confounders, the increase of trigger-day P did not affect the D6 available blastocyst rate. Although there was no significant difference in the D5 available blastocyst rate among different trigger-day P level groups, after adjusting for confounders, it was shown that the trigger-day elevated P (>1.5ng/ml), not the trigger-day slightly elevated P (1.0-1.5ng/ml) would lead to a decrease in the D5 available blastocyst rate. In the subgroup analysis of female age (< 35 vs ≥35 years) and insemination type (c-IVF vs ICSI), it was concluded that when the trigger-day P was more than 1.5ng/ml, the adjusted D5 available blastocyst rate would decrease significantly in two subgroups of women younger than 35 years and ICSI cycles. To our knowledge, few studies to date had evaluated whether trigger-day P affect available blastocyst rate. Only a retrospective analysis by Racca et al. (19) reported 3400 ICSI cycles with GnRH antagonist protocol for COS. Grouping by trigger-day P values (≤0.50, 0.51-1.49, ≥1.50 ng/ml), and their results showed that the available blastocyst rate on day 5 decreased with the increase of trigger-day P (48.8%, 47.8%, and 38.8%, respectively). However, the P cut-off values and detection method of Racca et al. (19) were different from those in our study, and the study of Racca et al. only included ICSI cycles, only involved D5 available blastocyst rate, did not analyze D6 available blastocyst rate and adjust confounders.

Our data showed that in c-IVF/ICSI cycles, the trigger-day P level was irrelevant to top-quality blastocyst rate. A single-center retrospective cohort study by Turgut et al. (30) in 2020 included 1485 ICSI cycles with GnRH antagonist protocol for COS. They stratified according to serum P levels (< 0.8 ng/ml; 0.8-1.49 ng/ml; ≥1.5 ng/ml). Generalized estimating equations (GEE) analysis also did not show that the effect of P levels on top-quality blastocyst rate had any statistical significance [OR, 1.07; 95%CI, 0.98-1.16; P=0.113; OR, 0.93; 95%CI, 0.80-1.07; P=0.32]. In 2023, Li et al. (11) recruited 504 eligible patients who underwent PGT for retrospective analysis, and grouped according to trigger-day P levels (cut-off values were 0.5 and 1.5 ng/ml, respectively) to assess the effect of trigger-day P on embryo quality. Their results showed that there was no significant difference in top-quality blastocyst rate (8.71% vs. 8.24% vs. 7.94%) among different P levels (P>0.05). These findings are consistent with our conclusion. However, all patients included in Li et al. (11) adopted a gonadotropin-releasing hormone agonist long-acting protocol and underwent PGT cycles (aneuploid blastocysts were excluded). Moreover, all D3 available embryos were cultured to D5 or D6. The P cut-off values of (0.5 and 1.5 ng/ml) were also different from those in our study. Confounding variables (such as female age) were also not adjusted. Although Turgut et al. (30) analyzed various confounding factors and made adjustments through GEE, the inclusion of only ICSI cycles may limit the overall application of their conclusions, and their definition of top-quality blastocyst rate was inconsistent with the present study. However, the above results that there was no correlation between trigger-day P level and top-quality blastocyst rate contradicted the studies of Kim et al. (6). In 2017, Kim et al. (6) found for the first time that the increase of trigger-day P in GnRH antagonist IVF/ICSI cycles was associated with a lower top-quality blastocyst rate. The reasons for the inconsistency between the findings of Kim et al. (6) and the conclusions of our study may be as follows: First, no embryos were transferred or frozen on D3 in the study of Kim et al. (6), all D3 available embryos were cultured to D5 or D6; Second, their definition of top quality blastocyst was also different from ours; Moreover, they statistically analyzed the top-quality blastocyst rate as a continuous variable, but, statistically, frequencies (such as top-quality blastocyst rate) should be described by categorical variables.

Animal studies had shown that lower P levels during the follicular phase can promote oocyte development *in vitro* (31). We conjecture that the high trigger-day P level delays embryo development or reduces embryo development potential by affecting oocyte quality, and large activation of the zygotic genome after the D3 embryonic phase cannot counteract the adverse effect of high trigger-day P level on embryo quality, leading to a decrease in D5 available blastocyst rate. However, the molecular mechanism by which high trigger-day P level affect blastocyst quality remains unclear. In addition, the underlying mechanism that causes trigger-day higher P level during ART is also unknown. Studies had shown that the number of follicles, the FSH drive and the LH activity were the three main factors that lead to increased P concentration during COS (32). According to the present study and earlier studies (13, 15, 20), trigger-day high P is usually accompanied by high E_2_. We also noticed that with the increase of trigger-day P, the female BMI gradually decreased, and the number of AFC and dominant follicles gradually increased.

All patients in this single-center study received a uniform ovarian stimulation protocol. Morphological assessment of blastocyst was performed by an experienced embryologist under equivalent laboratory conditions. Multiple regression logic analysis model was also used to explain the influence of various confounding factors on blastocyst culture outcomes, and subgroup analysis was performed according to female age and insemination type to ensure the reliability of the results.

However, due to the limitations of retrospective study, there were inevitable biases even if the impact of potential confounders was minimized. First, we used c-IVF/ICSI as a model, and the results cannot be extrapolated to all infertile populations. Second, trigger-day P does not have a definite cut-off value, which varies between studies at different centers and may vary depending on the detection method.

In summary, we used the c-IVF/ICSI cycles as a model to determine the relationship between trigger-day P and blastocyst culture outcomes. The results showed that the trigger-day slightly elevated P (1.0-1.5ng/ml) was not related to blastocyst culture outcomes, while the trigger-day elevated P (>1.5ng/ml) was an important factor affecting D5 available blastocyst rate, especially when the woman was younger than 35 years old or insemination type was ICSI. Because this study included c-IVF/ICSI patients treated with GnRH antagonist, it was unclear whether our findings can be extrapolated to populations using other protocols or to all infertility population. On the other hand, due to the small sample size and retrospective analysis in this study, the existence of bias cannot be ruled out, so it is recommended to carry out a randomized controlled trial with a larger sample size. In conclusion, in clinical practice, we should treat this result with caution. In order to predict the blastocyst culture outcomes, each center needs to evaluate the P threshold according to its own detection method when selecting D3 embryos for blastocyst culture.

## Data Availability

The raw data supporting the conclusions of this article will be made available by the authors, without undue reservation.
